# Insights into the function of the chloroplastic ribosome‐associated GTPase high frequency of lysogenization X in *Arabidopsis thaliana*

**DOI:** 10.1002/pld3.559

**Published:** 2024-01-11

**Authors:** Marwa Mehrez, Cécile Lecampion, Hang Ke, Faten Gorsane, Ben Field

**Affiliations:** ^1^ Aix‐Marseille Univ, CEA, CNRS, BIAM, UMR7265 Marseille France; ^2^ Laboratory of Molecular Genetics, Immunology and Biotechnology, Faculty of Sciences of Tunis University of Tunis El Manar Tunis Tunisia; ^3^ Faculty of Sciences of Bizerte University of Carthage Zarzouna Tunisia

**Keywords:** Arabidopsis, chloroplast, GTPase, HflX, stress, translation

## Abstract

Ribosome‐associated GTPases are conserved enzymes that participate in ribosome biogenesis and ribosome function. In bacteria, recent studies have identified HflX as a ribosome‐associated GTPase that is involved in both ribosome biogenesis and recycling under stress conditions. Plants possess a chloroplastic HflX homolog, but its function remains unknown. Here, we characterized the role of HflX in the plant *Arabidopsis thaliana* . Our findings show that HflX does not affect normal plant growth, nor does it play an essential role in acclimation to several different stresses, including heat, manganese, cold, and salt stress under the conditions tested. However, we found that HflX is required for plant resistance to chloroplast translational stress mediated by the antibiotic lincomycin. Our results suggest that HflX is a chloroplast ribosome‐associated protein that may play a role in the surveillance of translation. These findings provide new insight into the function of HflX as a ribosome‐associated GTPase in plants and highlight the importance of investigating conserved proteins in different organisms to gain a comprehensive understanding of their biological roles.

## 1 INTRODUCTION

Chloroplasts are the organelles in plant and algal cells responsible for photosynthesis, the process that fuels plant growth and most life on earth by converting sunlight into chemical energy. Chloroplasts also host several other critical metabolic pathways, including de novo lipid biosynthesis, nitrogen and sulfur fixation, and hormone synthesis. In addition to playing important roles for their hosts, chloroplasts are also a major nutrient resource, containing a significant portion of the plant's nitrogen and protein content, with Rubisco alone accounting for almost half of the soluble protein (Eckardt et al., 1997; Makino & Osmond, 1991).

Chloroplasts evolved from a symbiotic relationship between a cyanobacterium and a eukaryotic cell and have semi‐autonomous features, including their own genome and gene expression machinery. The chloroplast translation machinery is well studied and strongly resembles that found in bacteria, with the addition of chloroplast specific proteins (Zoschke & Bock, 2018). In bacteria, a suite of ribosome associated and translational GTPases assists bacterial ribosome assembly, translation, and ribosome turnover. Plant orthologs of these GTPases have been identified, although for many their molecular roles are poorly characterized (Mehrez et al., 2023; Suwastika et al., 2014). Certain are known to be essential, such as *SUPPRESSOR OF VARIEGATION11*, a plant homolog of the translation GTPase elongation factor TU (EF‐TU) (Liu et al., 2019), and ObgC, a plant homolog of the bacterial ribosome‐associated GTPase Obg (Bang et al., 2012; Chigri et al., 2009). The ribosome‐associated GTPase high frequency of lysogenization X (HflX) has recently received attention in both prokaryotes and animals. HflX binds to 50S ribosomal subunits (Jain et al., 2009), is implicated in ribosome biogenesis (Schaefer et al., 2006), and also acts as a ribosome splitting factor (Coatham et al., 2016; Dey et al., 2018; Rudra et al., 2020; Zhang et al., 2015). While not essential for growth under standard conditions in *E. coli*, HflX is required for acclimation to heat stress where its ribosome splitting activity allows recycling of stalled ribosomes (Dey et al., 2018; Zhang et al., 2015). Interestingly, in addition to its GTPase activity, HflX may also use an ATP‐dependent RNA helicase activity for the repair and reactivation of heat‐damaged ribosomal RNA (Dey et al., 2018). Animals possess an HflX ortholog known as GTPBP6. Like HflX, GTPBP6 is a ribosome recycling factor and is required for the assembly of mitochondrial ribosomes (Hillen et al., 2021; Lavdovskaia et al., 2020). In contrast to animals and bacteria, no role has been attributed to the plant HflX. However, Arabidopsis is reported to have a single *HFLX* gene, and the protein shows a chloroplastic localization (Suwastika et al., 2014) and is found in association with the 50S subunit of the chloroplast ribosome (Olinares et al., 2010).

In this study, we focus on the physiological function of the plant HflX. We show that the canonical HflX shares strong structural conservation with bacterial HflX enzymes. We also report a second non‐canonical plant HflX‐like enzyme that has independent evolutionary origins. With the use of independent T‐DNA insertion mutants, we show that the canonical HflX is dispensable for normal growth and development. Although HflX seems not to be involved in acclimation to a range of stress conditions, we find that it is required for resistance to lincomycin, an antibiotic that inhibits chloroplast translation. On the basis of this, we suggest that HflX is able to protect translation machinery either via blocking lincomycin binding to the ribosome or promoting the recycling of ribosomes stalled by lincomycin. Altogether, our results suggest that HflX is a chloroplast ribosome‐associated enzyme that plays a role in chloroplast translation, while its precise contribution to plant growth and stress acclimation remains uncertain.

## 2 MATERIAL AND METHODS

### 2.1 Plant material and growth conditions

The wild type was Col‐0. SALK_002001C (*hflx1‐1*), SALKseq‐041831.1 (*hflx1‐2*), and SALK‐057030.1 (*hflx1‐3*) were provided by the Signal Insertion Mutant Library (http://signal.salk.edu) (Alonso et al., 2003). Homozygous insertion mutants were isolated and confirmed by polymerase chain reaction (PCR).

For growth in normal conditions, seeds were sown in soil and transferred to separate pots 7 days after germination. Plants were grown either under long day conditions (16 h light/8 h dark) or short day conditions (8 h light/16 h darkness), at 22/18°C with 120 μmol photons m^−2^ s^−1^ lighting. For growth in culture dishes, seeds were surface sterilized in 70% ethanol containing 1% sodium hypochlorite and .005% Tween 20 for 10 min, then washed with 100% ethanol, dried and transferred in a grid pattern onto square plates containing 50 ml of MS/2 medium (.5× Murashige and Skoog salts [Merck Sigma‐Aldrich], 1% sucrose, .5 g/L MES, and .8% agar, adjusted to pH 5.7 with KOH). After 2 days of stratification at 4°C, plates were placed in a culture room with 16 h light (at 22°C)/8 h darkness (at 19.5°C) and 80 μmol photons m^−2^ s^−1^ lighting.

### 2.2 Stress treatments

We used the growth conditions mentioned above unless otherwise indicated. For heat stress, seedlings were grown on MS/2 for 12 days in standard conditions. Plates were transferred in a Percival and exposed to heat treatment for 24 h at 40°C, then, allowed to recover in standard conditions.

For manganese stress, seedlings were grown on MS/2 (with .4% phytagel [Sigma‐Aldrich] instead of .8% agar) supplemented or not with 2‐mM filter sterilized MnSO_4_.

For cold stress, seeds were grown on MS/2 for 7 days in standard conditions with 50‐μmol photons m^−2^ s^−1^ lighting. Plates were then either kept in standard conditions (control) or transferred to a cold room at 5°C with 40 μmol photons m^−2^ s^−1^ lighting.

For salt stress, seedlings were sown on MS/2 without sucrose and grown for 7 days and then transferred to MS/2 without sucrose supplemented or not with 150‐mM NaCl.

For lincomycin treatment, seeds were germinated on MS/2 containing 35‐μM filter sterilized lincomycin.

### 2.3 Genotyping

DNA extraction was based on the approach of Edwards et al. (1991) with modifications. DNA from one leaf disk was extracted in 400 μl of DNA extraction buffer (200‐mM Tris‐HCl, 250‐mM NaCl, 25‐mM EDTA, .5% w/v SDS, 20 μg/ml RNAse, pH 7.5) using a pellet pestle in 1.5‐ml tube. The samples were incubated for 1 h at 65°C and centrifuged at 3000 ***g*** for 10 min. The supernatant was transferred to a new tube containing 200 μl of phenol‐chloroform‐isoamyl alcohol (25:24:1). Tubes were inverted several times, left for 5 min, and centrifuged at 3000 ***g*** for 10 min at 4°C. the upper aqueous phase was transferred to a new tube to which an equal volume of isopropanol was added. The tubes were then left for 1 h at room temperature. After centrifugation at 4000 ***g*** for 25 min, the supernatant was discarded, and the pellet was washed with 70% ethanol. The ethanol was completely removed, and the pellet was air dried and resuspended in TE buffer (10‐mM Tris‐HCl, 1‐mM EDTA, pH 8.0). T‐DNA insertions were then analyzed using specific primers (Table S1) in PCR reactions with Emerald Master Mix (Takara). PCR conditions were as follows: an initial step at 98°C for 30 s, followed by 38 cycles of 98°C for 15 s, 58°C for 20 s, and 72°C for 1–1 min 30 s.

### 2.4 RNA extraction, RT‐PCR, and qRT‐PCR

RNA extraction was performed using Tri‐Reagent (Sigma‐Aldrich) and quality confirmed by agarose gel‐electrophoresis. RNA was treated with DNAse I (Thermo scientific), and cDNA was synthesized from 500 ng of RNA using Primescript RT Reagent Kit (Takara) with random hexamer primers. RT‐PCR was performed as mentioned above for genotyping reactions using specific primers (Table S1). qRT‐PCR was carried out in a Bio‐Rad CFX96 real‐time system using the following conditions: 95°C for 30 s, followed by 44 cycles of 95°C for 5 s, 59°C for 30 s, and 72 °C for 30 s. Each qRT‐PCR reaction was performed in 15‐μl reaction volume that consisted of 1 μl of cDNA (12.5 ng/μl), 2.4 μl of primer mixture (2.5 μM for each primer) (Table S1), and 7.5‐μl TB Green Premix Ex Taq II (Tli RNaseH Plus) (Takara). Melting curves were performed to confirm amplification specificity.

### 2.5 Plant growth measurements

For rosette area measurements, plants grown on soil were photographed at different times during their growth using a camera (Panasonic Lumix, 20‐1200). Images were then automatically analyzed using the ARADEEPOPSIS pipeline (Hüther et al., 2020). For measurements of seedling area, plates were scanned at the indicated time. The images were then analyzed in ImageJ (NIH) and the area from each seedling was obtained.

### 2.6 Chlorophyll fluorescence analysis

Chlorophyll fluorescence was measured in a Fluorcam FC 800‐O imaging fluorometer (Photon System Instruments). The plants were adapted to dark for 20 min, and the PSII maximum quantum yield (Fv/Fm) was calculated as (Fm − Fo)/Fm.

### 2.7 Chlorophyll quantification

Chlorophyll quantification was performed as previously described (Sugliani et al., 2016). Briefly, chlorophyll was extracted from frozen seedlings homogenized using pellet pestle in ice cold 90% acetone saturated with sodium carbonate and kept overnight at −20°C. When the plant material was completely white, the samples were centrifuged, and the supernatant was transferred to a new tube. The absorbance was measured between 350–750 nm using an 80% acetone blank in a Varian Cary 300 spectrophotometer (Agilent). Total chlorophyll content was calculated using a full spectra fitting algorithm (Chazaux et al., 2022). For each line, 5 biological samples from different plates were used for the absorbance measurements. The experiment was repeated twice.

### 2.8 Phylogenetic inference and protein structure analysis

Using *E. coli* HflX as a query, homologous proteins from photosynthetic organisms were identified by BLAST search using public data at the National Center for Biotechnology Information (NCBI) and JGI. The representative bacterial HflX/HflXr and animal homologs were previously identified (Koller et al., 2022; Suwastika et al., 2014). Multiple‐sequence alignments were performed using MAFFT v7.40262 with option "auto" (Katoh et al., 2019). Phylogenetic reconstructions were created using maximum likelihood with the IQ‐TREE web server version 1.6.12 using default settings, with LG + F + I + G4 automatically selected as the best fit evolutionary model based on BIC values by ModelFinder (Trifinopoulos et al., 2016). Branch support was tested using two methods: ultrafast bootstrap approximation using 1000 bootstraps and the non‐parametric Shimodaira–Hasegawa‐like approximate likelihood‐ratio test (aLRT). The alignments and trees are available in supplementary Data S1.

For structural analysis, protein structures were retrieved from RSCB Protein Data Bank (PDB) or the EMBL‐EBI Alphafold database (Jumper et al., 2021; Varadi et al., 2022) and visualized using ChimeraX (Pettersen et al., 2021).

### 2.9 Data analysis

Data analysis and visualization was conducted in R using scripts previously described (Romand et al., 2022) with minor modifications. Data generated from ARADEEPOPSIS were analyzed using the script provided in https://github.com/cecile-lecampion/Analyse_croissance. For each experiment, plates were considered as independent replicates, and individual plants were considered as biological replicates. The experiments were performed at least twice, and similar results were obtained. The significance of differences in categorical data (cotyledon death) was calculated using the proportion test as previously described (Romand et al., 2022).

## 3 RESULTS

### 3.1 *Arabidopsis thaliana* contains two HflX homologs with different evolutionary origins

**FIGURE 1:**
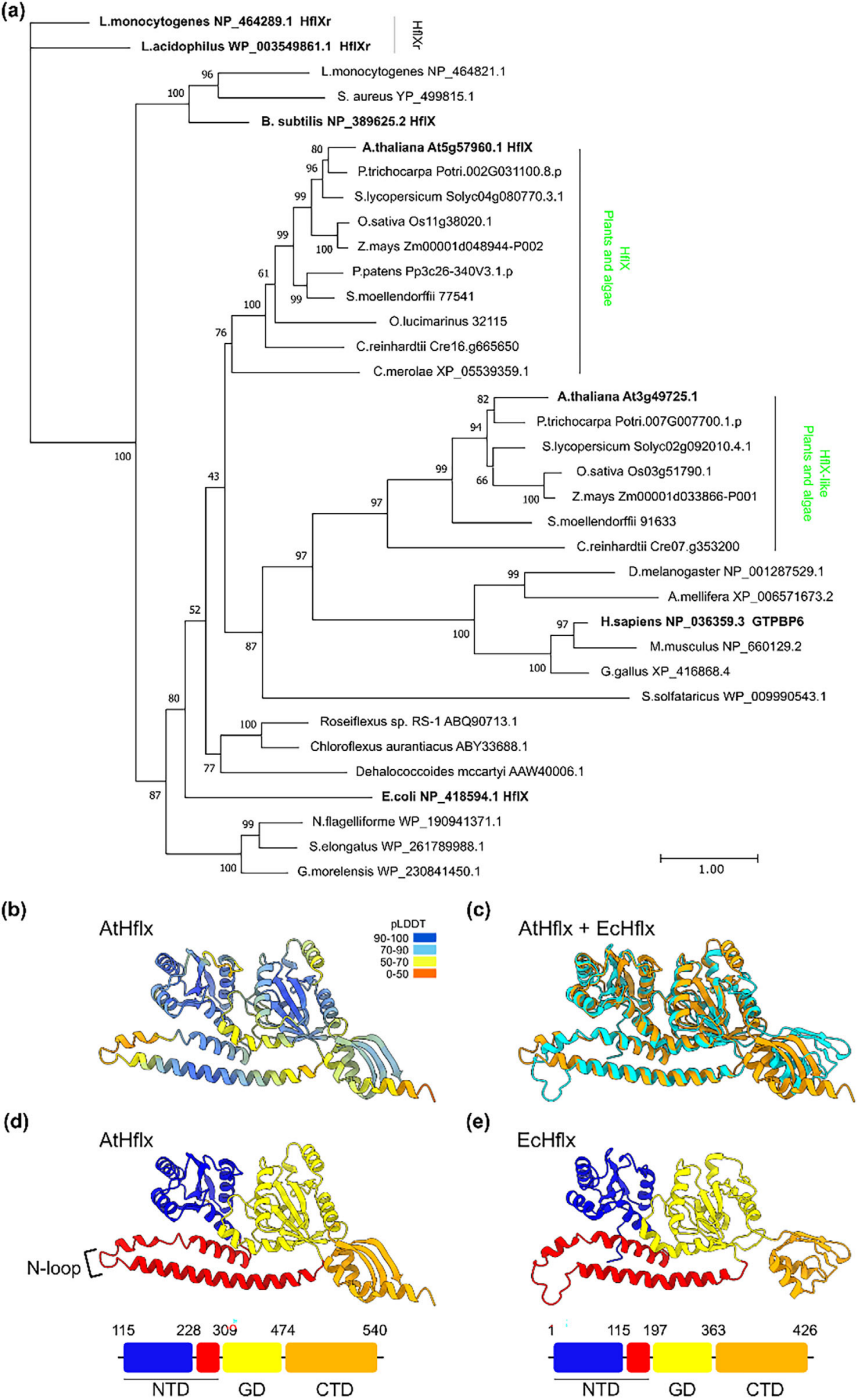
Arabidopsis contains an HflX homolog with a conserved structure. (a) A maximum‐likelihood phylogenetic tree of selected HflX proteins from eukaryotes and prokaryotes. Plant and algal HflX clades are indicated. The scale bar indicates substitutions per site, and statistical support for branches is shown at the nodes. (b) Alphafold model of Arabidopsis HflX (AtHflX, Q9FJM0) with confidence‐per‐residue coloring (pLDDT). The predicted chloroplast transit peptide is not shown. (c) Arabidopsis HflX model (orange) aligned with the structure of ribosome‐associated *E. coli* HflX (cyan) (PDB 5ADY)(Zhang et al., 2015). Domain organization of the (d) Arabidopsis and (e) *E. coli* HflX enzymes. NTD, N‐terminal domain containing two subdomains (nucleotide binding domain 1 in blue, helical domain in red); GD, G‐domain (nucleotide binding domain 2); CTD, C‐terminal domain.

We analyzed the distribution of HflX enzymes in plants, bacteria, and other organisms. We found that the majority of green plants possess two HflX homologs, encoded by single copy genes (Figure 1a). Phylogenetic analysis confirmed the previously identified HflX groups with the green non‐sulfur bacteria rather than cyanobacteria (Suwastika et al., 2014). This plant and algal HflX clade includes an HflX from the unicellular red alga *Cyanidioschyzon merolae*, strongly suggesting an evolutionary origin close to the emergence of chloroplast‐containing organisms. Surprisingly, we found a second group of plant HflX‐like enzymes that forms a separate clade with distinct evolutionary origins. Interestingly, this plant HflX‐like clade appears to be closely related to the animal HflX enzymes, as well as an HflX from the thermophilic archaeon *Sulfolobus solfataricus*. Interestingly, we note that the plant HflX‐like enzymes are not canonical because they lack the conserved C‐terminal domain (CTD) found in animal and bacterial HflX enzymes (Figure S1).

Recently, HflXr enzymes required for enhanced antibiotic resistance were discovered in *Listeria monocytogenes* bacteria (Duval et al., 2018; Koller et al., 2022). Our phylogenetic analysis shows that plants and algae clearly lack HflXr orthologs.

Next, we analyzed the Alphafold predicted protein structure of the canonical Arabidopsis HflX (Figure 1b). Arabidopsis HflX displays the same domain organization as the *E. coli* HflX with the presence of a conserved N‐terminal domain (NTD), G domain (GD), and CTD (Figure 1d,e). The NTD and GD showed strong similarities at the structural level when compared with the ribosome‐bound *E. coli* HflX, with the exception of a small alpha‐helix extension in the GD (Figure 1c). The NTD of *E. coli* HflX interacts with the 23S rRNA on the 50S subunit. It is therefore likely that Arabidopsis HflX is able to interact with the 50S ribosomal subunit in a similar fashion to the *E. coli* HflX. The CTD showed more differences, with an altered orientation and an additional alpha‐helix. Residues considered important for ATPase (corresponding to *E. coli* HflX Arg90 and Asp102) and GTPase activity (Gly252 and Ser343) are also conserved (Lavdovskaia et al., 2020) suggesting that the Arabidopsis HflX may be able to perform both ribosome splitting and helicase functions. *E. coli* HflX also possesses an autophosphorylation activity on Ser211 (Ghosh et al., 2016). The corresponding residue is conserved in AtHflX. Both the sequence and structural conservation suggest that the canonical Arabidopsis HflX is therefore able to play a similar role to bacterial HflX enzymes.

Despite belonging to the HflX family, the plant HflX‐like enzymes show major differences with respect to canonical HflX enzymes (Figure S1). Arabidopsis HflX‐like possesses a conserved NTD and GD core, yet lacks a C‐terminal region resembling the HflX CTD. This is instead replaced with an unstructured tail. In addition, there is an enlarged loop (N‐loop) in the NTD. In *E. coli* HflX, the N‐loop extends into the peptidyl‐transferase center (Zhang et al., 2015). The enlarged N‐loop of the HflX‐like NTD is therefore likely to profoundly alter the manner in which the enzyme is able to interact with ribosomes. Finally, the localization of HflX‐like is not yet resolved. Indeed, Target P predicts a chloroplastic localization (likelihood = .63) (Almagro Armenteros et al., 2019), and the protein itself was identified in mitochondrial ribosome fractions (Rugen et al., 2019).

### 3.2 The canonical HflX is not essential for growth under standard conditions

**FIGURE 2:**
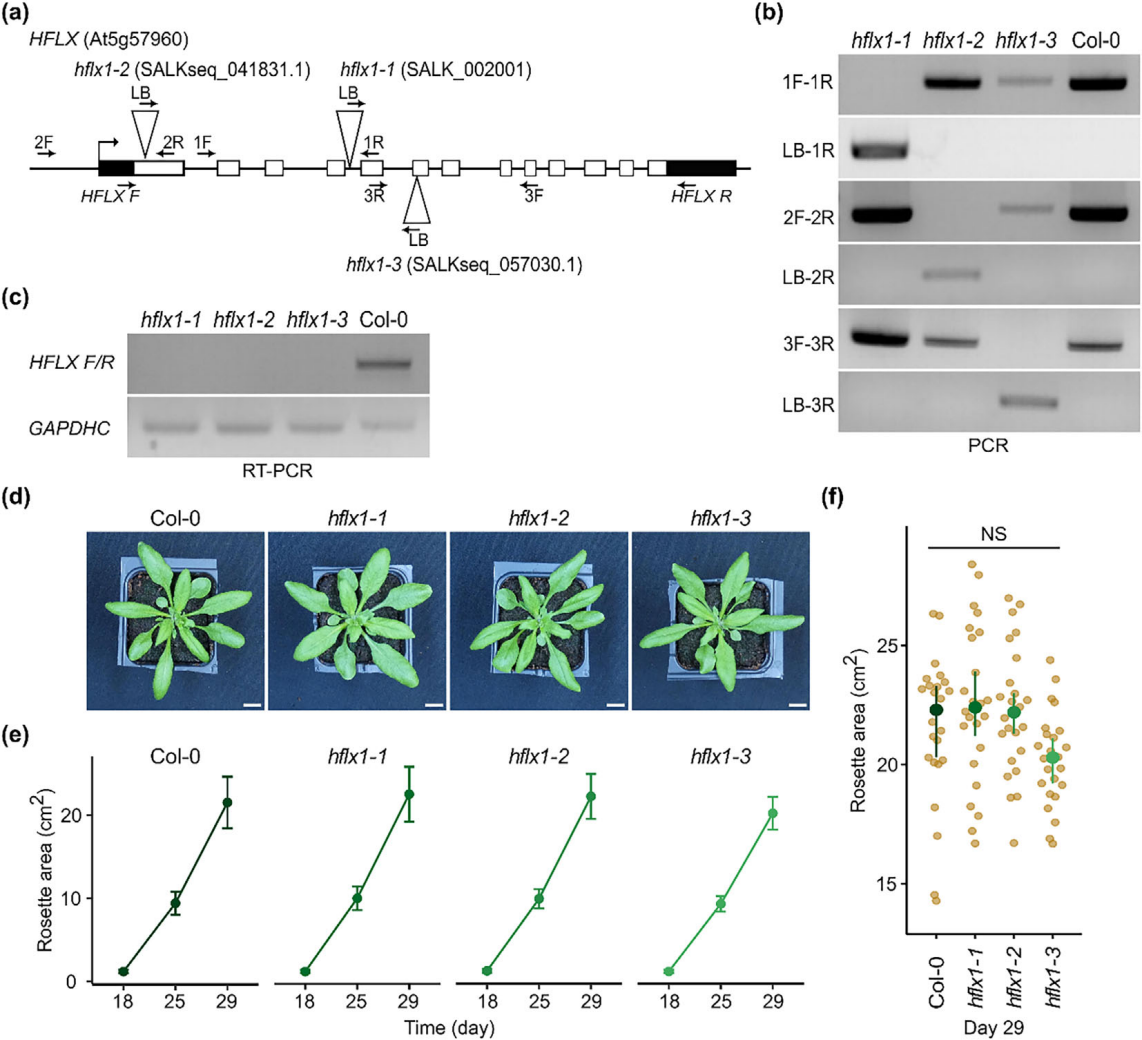
HflX is not essential for vegetative growth in Arabidopsis. (a) Localization of T‐DNA insertions in the canonical *HFLX* gene. Arrows indicate the position of genotyping primers. (b) Genotyping of *hflx* mutants using primers shown in (a). (c) reverse transcription polymerase chain reaction (RT‐PCR) amplification of the full‐length *HflX* cDNA in the wild type Col‐0 and the three *HflX* insertion mutants. (d–f) The phenotype of wild type (Col‐0) and *hflx* mutants grown in long day conditions. (d) Photographs of plant rosettes at Day 29, (e) quantification of vegetative growth rates, and (f) comparison of rosette area at Day 29 (*n* = 24 plants per genotype). Scale bar, 1 cm. Graphs show mean and 95% CI. NS, not significant.

To identify the role of the canonical *HFLX* in plants, we analyzed three *HFLX* T‐DNA insertion mutants that we named *hflx 1‐1*, *hflx 1‐2*, and *hflx 1‐3* (Figure 2a). We isolated homozygous lines (Figure 2b) and confirmed the absence of a full‐length *HFLX* transcript in the three mutants (Figure 2c). This indicates that the three mutants are unable to produce the full length protein and are likely to be loss‐of‐function knockouts.

To examine whether *HFLX* is required for plant growth, *hflx* mutants and the wild type Col‐0 were grown in standard conditions, and the growth rate was quantified. The three mutants showed no significant different in rosette size from the wild type under long day conditions (Figure 2d–f). The results were similar in short day conditions, with the exception of *hflx 1‐3* that was significantly smaller than all the other lines at Day 39 (Figure S2). As the *hflx 1‐3* phenotype was not observed in the other mutants, it cannot be explained by the knockout of *HFLX*. We conclude that HflX is not essential for normal vegetative growth in Arabidopsis.

### 3.3 HflX does not play a major role in resistance to a range of abiotic stresses

**FIGURE 3:**
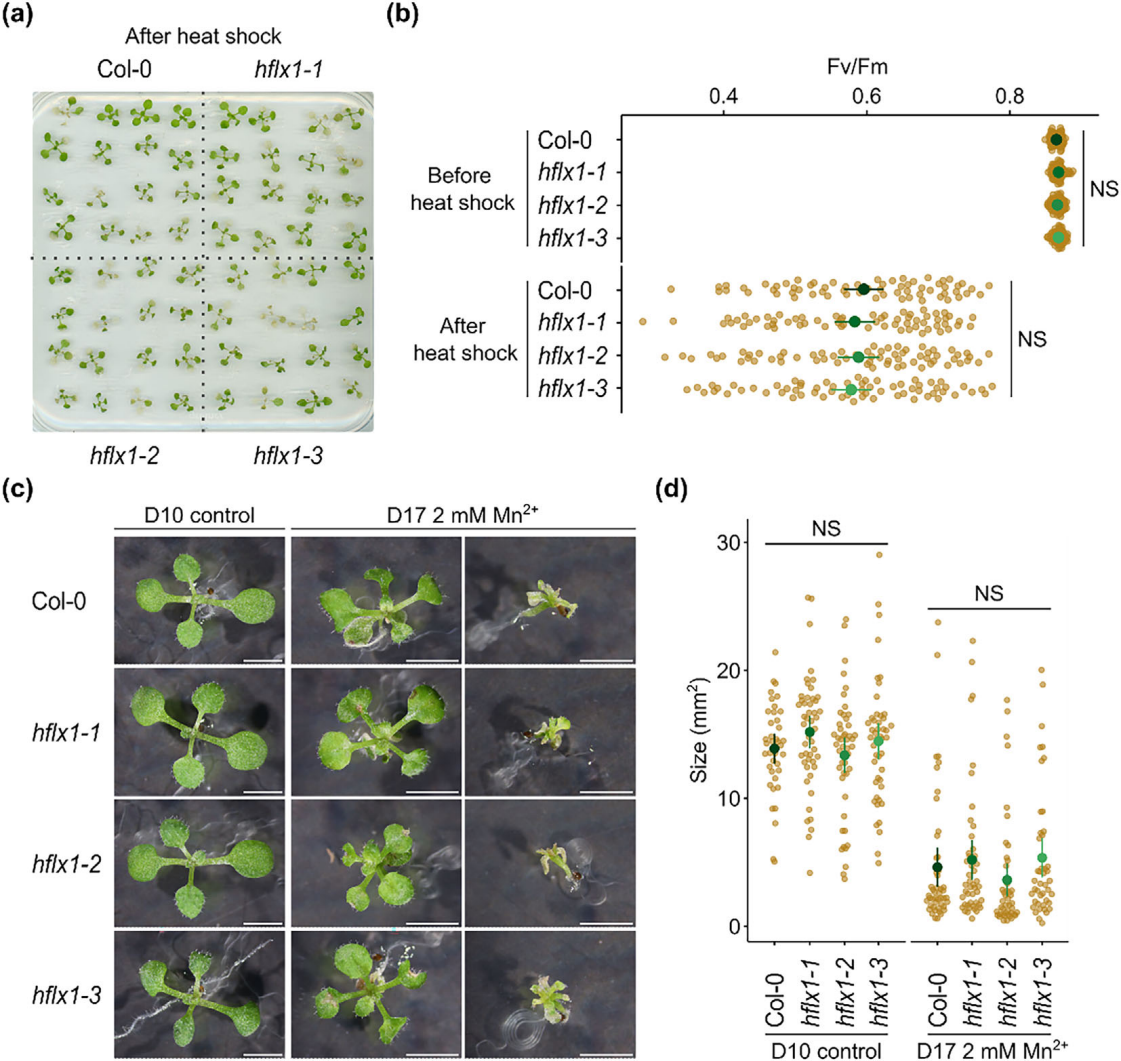
HflX does not appear to be involved in acclimation to heat shock or excess manganese. (a) 12‐day‐old seedlings were subjected to a heat shock treatment for 24 h at 40°C and photographed after 1 day of recovery. (b) PSII maximal efficiency (Fv/Fm) was measured in seedlings before and after heat shock treatment, *n* = 64 plants per genotype. (c) Seedlings were grown on medium supplemented or not with manganese and photographed at the indicated day. Scale bar, 3 mm. (d) Comparison of plant size of seedlings grown on medium with or without manganese at the indicated day. *n* = 44–47 plants per genotype. Graphs show mean and 95% CI. NS, not significant.

In *E. coli*, the *hflx* mutant is hypersensitive to heat shock (HS) (Zhang et al., 2015), and HflX exhibits an ATP‐dependent helicase activity that is necessary for RNA unwinding and rescuing heat‐damaged 50S subunits (Dey et al., 2018). The structural similarity between *E. coli* and Arabidopsis HflX might suggest conservation of a role in acclimation to heat‐shock. Therefore, we investigated the resistance of the three *hflx* mutants to a HS treatment at 40°C for 24 h. One day after the HS, *hflx* mutants and wild type plants became pale green and showed evidence of cotyledon death (Figure 3a). The efficiency of photosystem II (Fv/Fm) also decreased in response to HS, although there was no difference between the *hflx* mutants and the wild type (Figure 3b). These results revealed that Arabidopsis HflX is not required for acclimation to HS under the conditions tested.

In *E. coli*, *hflx* mutants are also hypersensitive to excess manganese, a stress characterized by growth arrest, filamentation, and lower rates of replication (Kaur et al., 2014; Sengupta et al., 2018). Therefore, we investigated the effect of excessive manganese on the growth of *hflx* mutants by germinating seeds on a medium supplemented with 2‐mM Mn^2+^ or on control plates without manganese. After 10 days, the untreated seedlings showed a similar phenotype to the wild‐type control Col‐0 (Figure 3c). *hflx1‐1* mutant seedlings were slightly bigger than the wild type and other mutants; however, this difference was not significant (Figure 3d). Excess manganese caused a heterogenous response among all the lines tested. Some seedlings were only mildly affected, whereas others showed severe growth limitation, chlorophyll loss and cotyledon death (Figure 3c). No significant difference was observed between lines after 17 days of treatment (Figure 3d).

Next, we investigated the effect of cold and salt stress, two stresses known to perturb chloroplast gene expression (Hao et al., 2021; Zoschke & Bock, 2018). For cold stress, seedlings were transferred to 5°C and grown for 4 weeks (Figure S3a). Growth was greatly inhibited during the first week and then resumed. The efficiency of photosystem II (Fv/Fm) showed a slight drop after cold stress treatment for all lines (Figure S3b). However, no significant differences were detected between the mutants and the wild type. For salt stress treatment, seedlings were transferred to a medium supplemented with 150‐mM NaCl. Four days after transfer, many seedlings became pale and showed evidence of cotyledon death. A similar phenotype was observed for both the wild type and the *hflx* mutants (Figure S3c). Although *hflx1‐1* seedlings showed a higher rate of cotyledon death, no significant difference was observed between lines (Figure S3d). Overall, we found that HflX does not appear to be required for acclimation to heat, manganese, cold, or salt stress under the conditions tested. Therefore, HflX does not appear to play a major role in acclimation to these stresses, although we cannot completely exclude a role under specific circumstances.

### 3.4 *hflx* mutants are sensitive to lincomycin

**FIGURE 4:**
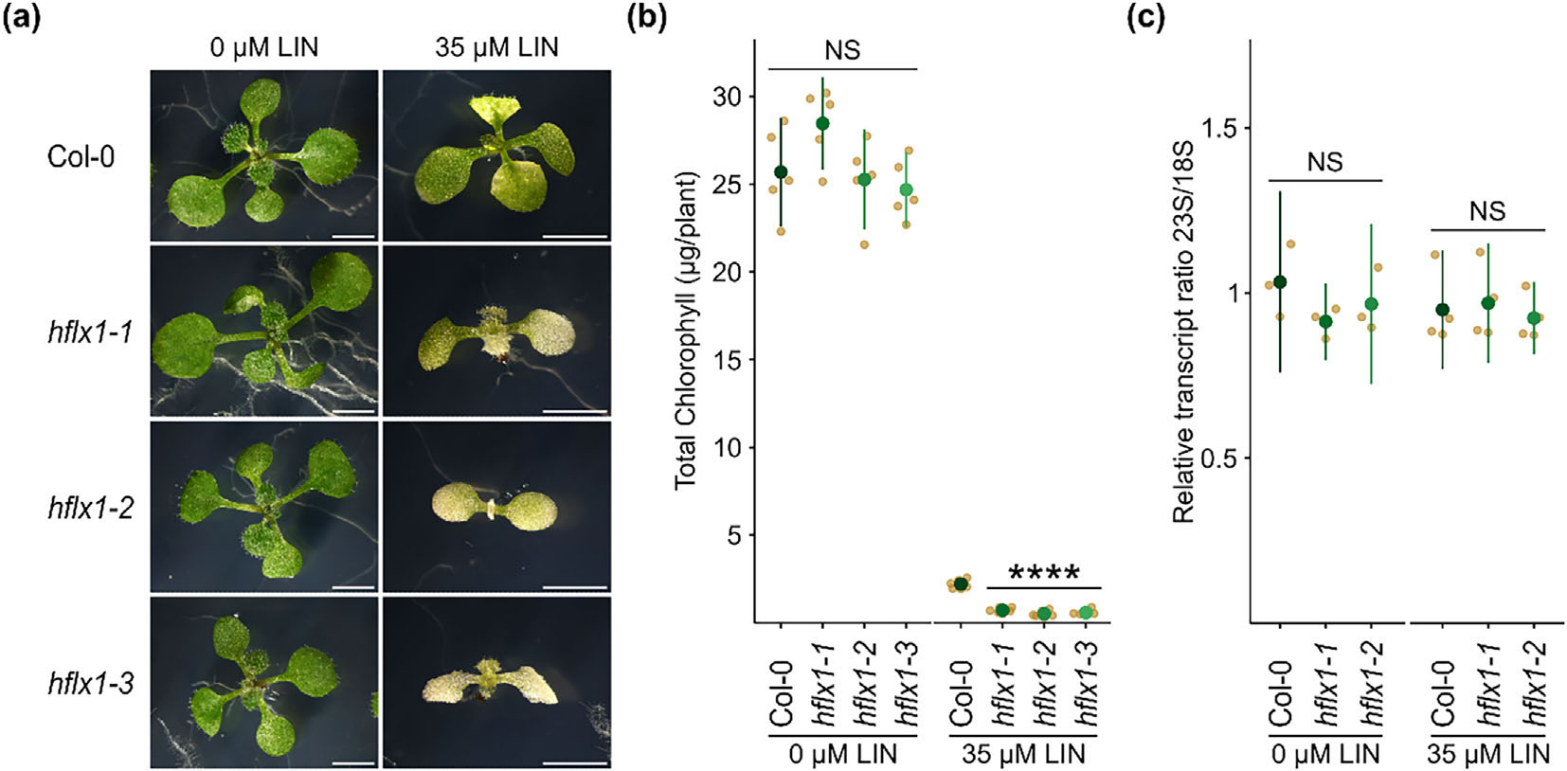
HflX is required for resistance to the antibiotic lincomycin. (a) Seedlings were grown on medium with or without lincomycin and photographed after 12 days. Scale bar, 3 mm. (b) Chlorophyll content was quantified in 15‐day‐old seedlings, *n* = 5 biological replicates. (c) Ratio of the chloroplast 23S rRNA to the cytoplasmic 18S rRNA in Col‐0, *hflx1‐1*, and *hflx1‐2*. Quantitative reverse transcription polymerase chain reaction (qRT‐PCR) was performed on cDNA extracted from seedlings grown on medium with or without lincomycin for 12 (untreated) or 15 (treated) days. *n* = 3–4 independent biological replicates. Graphs show mean and 95% CI. NS, not significant.

Next, we used lincomycin to directly inhibit chloroplast translation in seedlings. Strikingly, we found that the three *hflx* mutants were clearly more sensitive to lincomycin than the wild type (Figure 4a). Lincomycin sensitivity was further confirmed by quantifying chlorophyll content, which was significantly lower in the *hflx* mutants than in the wild‐type control (Figure 4b).

To determine whether the *hflx* lincomycin sensitivity was due to changes in chloroplast rRNA levels, we quantified the ratio of chloroplast 23S rRNA to cytosolic 18S rRNA by qPCR (Figure 4c). The 23S/18S rRNA ratio was similar between Col‐0, *hflx1‐1*, and *hflx1‐2* under both control and lincomycin treatment conditions and showed no significant differences. Therefore, *hflx* does not appear to be required for the build‐up of chloroplast rRNA levels under control or stress conditions.

## 4 DISCUSSION

Recently, it was reported that two HflX homologs, HflX and HflXr, are found in bacteria (Duval et al., 2018; Koller et al., 2022). Our analyses confirmed that the HflX of green plants and algae is closer to HflX than HflXr (Figure 1a). This is in line with the wider distribution of HflX and the specific association of HflXr with antibiotic resistance. In addition, our results reinforce previous findings suggesting that the plant HflX originated from green non‐sulfur bacteria through lateral gene transfer (Suwastika et al., 2014). Interestingly, we also found a second clade of plant HflX‐like enzymes that lacks the CTD that characterizes the majority of HflX enzymes in plants, bacteria, and animals (Figures 1a and S1). The CTD, which does not interface directly with the ribosome, is reported to be less conserved compared with the other domains. Indeed, the CTD is also absent from the HflX of the archaeon *S. solfataricus* (Wu et al., 2010).

We show that the canonical HflX is not required for vegetative plant growth under normal conditions (Figures 2 and S2). While we did not observe any other obvious phenotypic differences, we cannot exclude the possibility that HflX is required for other aspects of growth and development not tested here. Nevertheless, our results appear rather similar to those for the *E. coli* HflX that is dispensable for normal growth (Zhang et al., 2015). In contrast, GTPBP6, the human ortholog of HflX, is essential for cell survival and gene expression under physiological conditions (Lavdovskaia et al., 2020). This is likely due to the essential role of GTPBP6 in the assembly of mitochondrial ribosomes.

The *E. coli* HflX possesses an ATP‐dependent RNA helicase activity in the NTD that has an essential role in restoring heat damaged ribosomes (Dey et al., 2018). Although this domain is conserved in Arabidopsis HflX, with conservation of structure and essential residues for ATPase activity, Arabidopsis *hflx* mutants did not display compromised heat tolerance (Figure 3). Similarly, we were unable to detect increased sensitivity to excess manganese, another phenotype observed in *E. coli* HflX mutants (Kaur et al., 2014; Sengupta et al., 2018). This suggests that Arabidopsis HflX is not involved in chloroplast ribosome rescue or manganese homeostasis under the conditions tested or that HflX plays a redundant role that can be replaced by other factors. One possibility is that HflX‐like, which possesses the necessary domains, might also contribute to heat and manganese stress acclimation. It would therefore be interesting to test the chloroplast localization of HflX‐like and investigate the phenotype of *hflx* and *hflx‐like* double mutants.

We also show that, in addition to heat and manganese stress, *hflx* mutants are not hypersensitive to cold or salt stress (Figure S3). These conditions are known to affect chloroplast function and in particular chloroplast translation (Hao et al., 2021; Hu et al., 2020; Zoschke & Bock, 2018). Indeed, some chloroplast translation factors are known to be required under such conditions (Li et al., 2018; Pulido et al., 2018). This would suggest that the removal of HflX does not perturb chloroplast ribosome biogenesis or translation enough to cause a detectable phenotype.

The hypersensitive phenotype we found in response to lincomycin treatment strongly implies that HflX is indeed ribosome‐associated and likely involved in the surveillance of chloroplast translation. However, even though HflX is not essential, it is still uncertain whether it participates in ribosome biogenesis. We did not observe a reduction in chloroplast rRNA levels in the *hflx* mutants. However, this does not exclude involvement in the later steps of ribosome biogenesis. For example, the human GTPBP6 mutant does not show alterations in the steady‐state levels of mitochondrial rRNAs or severe changes in the steady state levels of ribosomal proteins, yet still shows critical defects in mitochondrial large subunit assembly (mitoLSU) (Lavdovskaia et al., 2020). Lincomycin inhibits prokaryotic protein synthesis by interacting with the peptidyl transferase center (PTC). HflX and HflXr resistance to macrolide‐lincosamide antibiotics is conferred by their ability to split and recycle stalled ribosomes (Duval et al., 2018; Rudra et al., 2020). HflXr also employs a second resistance mechanism. The cryo‐EM structure of HflXr reveals that it binds analogously to *E. coli* HflX on the 50s subunit, with the N‐loop of the NTD positioned deeper within the PTC. Upon binding to the ribosome, HflXr induces conformational changes in the PTC that are incompatible with antibiotic binding (Koller et al., 2022). A similar mechanism was more recently observed for HflX mediated resistance to chloramphenicol in *E. coli* suggesting that it is not limited to HflXr (Wu et al., 2022). In line with this, and considering the structural differences of the chloroplast ribosome compared with the bacterial ribosome (Manuell et al., 2007; Sharma et al., 2007; Yamaguchi & Subramanian, 2000, 2003), the antibiotic sensitivity of *hflx* mutants might be due to better access of lincomycin to its binding site in the absence of HflX or loss of the capacity of HflX to recycle lincomycin stalled ribosomes. We note that, in either case, HflX‐like is not able to prevent lincomycin sensitivity. This may be because HflX‐like is localized in the mitochondria rather than the chloroplast (Rugen et al., 2019) or because the atypical structure of HflX‐like alters or even prevents ribosome binding.

In conclusion, our data suggest that Arabidopsis HflX is a conserved HflX ortholog that is associated with the chloroplast ribosome and is likely to play a role in the surveillance of chloroplast translation. Even so, Arabidopsis HflX seems not to be actively involved during stress acclimation, suggesting that it is likely redundant with other plant factors, or plays an unknown role. Our results highlight the challenges of exploring translation regulation within the chloroplast and further emphasize that while the functions of some ribosome‐associated proteins are evolutionary conserved between organelles and bacteria, others are likely to be organism‐dependent.

## AUTHOR CONTRIBUTIONS

FG and BF conceived and coordinated the project. MM, HK, and BF designed the experiments. MM and HK isolated the plant mutants. MM carried out the remaining experiments. MM and BF analyzed the data. CL assisted in data visualization and statistical analysis. MM, FG, and BF wrote the manuscript, and all authors commented and approved the final version of the manuscript.

## CONFLICT OF INTEREST STATEMENT

The authors did not report any conflict of interest.

## PEER REVIEW

The peer review history for this article is available in the Supporting Information for this article.

## Supporting information


**Figure S1.** Structure of Arabidopsis HflX‐like.
**Figure S2.** HflX is not essential for vegetative growth under short days.
**Figure S3.** HflX does not appear to be required for acclimation to cold or salt stress
**Table S1.** Primers
Supplementary file 1. Alignment and phylogeny inference files.

**Data S1.** Peer review

## Data Availability

The datasets, scripts, and materials used in the current study are available from the corresponding author on request.
