# Supplementary material for: Insights into the function of the chloroplastic ribosome‐associated GTPase high frequency of lysogenization X in Arabidopsis thaliana
Source: Plant Direct. 2024 Jan 11;8(1):e559. doi: 10.1002/pld3.559 (PMC10784650; doi:10.1002/pld3.559)
Supplement: Supplementary file 1 — Figure S1. Structure of Arabidopsis HflX‐like. Figure S2. HflX is not essential for vegetative growth under short days. Figure S3. HflX does not appear to be required for acclimation to cold or salt stress Table S1. Primers Supplementary file 1. Alignment and phylogeny inference files. [file PLD3-8-e559-s002.pdf]

Plant Direct
Open Access

Insights into the function of the chloroplastic ribosome-associated GTPase HflX in *Arabidopsis thaliana*.

Marwa Mehrez[1,2], Cécile Lecampion[1], Hang Ke[1], Faten Gorsane[2,3], Ben Field[1]

DECISION LETTER- ROUND 1

June 12, 2023

Dr. Ben Field
CNRS, CEA, University of Aix-Marseille
BIAM
163 av de Luminy
Marseille, N/A 13009
France

RE: Insights into the function of the chloroplastic ribosome-associated GTPase HflX in Arabidopsis thaliana.

Dear Dr. Field:

Thank you for submitting to Plant Direct. All required reviews have been returned and we have now finished our evaluation of your manuscript. In light of the reviewers' and editor's comments, further revisions are needed before the paper can be accepted for publication in Plant Direct.

Please view the editors' and reviewers' comments below and use their suggestions as a guide while you work on your revision.

When uploading the revised version of this article, please be sure to include the following:

-A word document that contains your response to the reviewers. You should respond to each reviewer comment and note the changes made to the manuscript. If you do not agree with a reviewer's comment and choose not to make a suggested revision, please explain why. Please try to provide as complete an answer as possible to each reviewer's criticisms.
-A tracked changes document with each change highlighted
- A clean version of the latest version of the manuscript

To upload your revision, please click the link below.
https://plantdirect.msubmit.net/cgi-bin/main.plex?el=A7Lr2Zo4A1gqn7I2A9ftdTsJrY7o7eQXKdn3HsdNcfQZ

In order to provide as timely a service as possible, we ask that your revision is resubmitted within three months after receipt of this request. If an extension is needed, please send a request, along with a brief explanation, to the editorial office at plantdirect@wiley.com .

Please note that, in addition to publishing reviewer comments, the author's responses to review comments will also be published alongside the final version of the paper. If you would not like the author's responses to be published, please contact the editorial office at plantdirect@wiley.com .

Thank you very much for giving us an opportunity to review your work. I look forward to receiving the next version.

Sincerely,

Ana Fortes

Editor, Plant Direct

-- Editor comments:

One reviewer noted the need for the authors to make sure their claims are supported by the data. This should be thoroughly revised in the next version. Though there is no need to provide additional experiments the statements have to be sound.

-- Reviewer comments:
Reviewer #2:

This manuscript reports initial efforts to gather information on the physiological function of the plant HflX protein. With incomplete approaches, the authors address the function of this protein by evaluating the phenotypes of Arabidopsis plants under different growth conditions, including heat, manganese, cold, and salt stress, where they did not detect differences between the null hflX mutants and the wild-type line. However, when they add lincomycin, an antibiotic that affects chloroplast translation, to the plant growth medium, hflX mutants showed resistance to this antibiotic as compared to the wild type. With no further analysis, authors conclude that "HflX does not have a detectable role in plant growth and development, nor does it play a distinct role in acclimation to several different stresses" and "that HflX is required for plant resistance to chloroplast translational stress mediated by the antibiotic lincomycin". Even though the described observations add information to the functional study of this protein in plants, further experimentation is needed to support the concluding statements of this work. Authors should consider the following observations.

Introduction
How many HflX genes are in Arabidopsis? Does the number of HflX and HflX-like genes vary between different plant species?

Results

Page 8. During the analysis of the structural characteristics of the Arabidopsis HflX and HflX-like proteins, did authors look for possible phosphorylation sites given the observation that E. coli HflX apparently possess an autophosphorylation activity (Gosh et al., doi: 10.1002/2211-5463.12065 FEBS 2016)

Page 9. After evaluating a rosette growth through the measurement of its size in wild type and mutant lines, authors concluded that HflX does not influence Arabidopsis normal growth and development. However, testing just this phenotype is not enough to reach such wide conclusion. Authors do not mention if they evaluated germination rate, leaf size, flowering time, seed size, yield, root architecture, etc, etc. Authors should assess additional phenotypes during different developmental stages to determine whether there is or not any effect on growth and development in Arabidopsis plants lacking HflX. Maybe checking those traits where an active translation is needed would a good start.

Page 11. Do authors compare basal heat tolerance of the mutants, or other phenotypes to look at the mutant performance through different heat plant responses? Do authors consider that the evaluation they did is enough to be certain that HflX does not participate in acclimation to HS?

Page 11. Regarding manganese effects, just one manganese concentration (2 mM) was tested for this evaluation? Why was 2 mM manganese used in these experiments? A manganese concentration sensitivity curve should be done including all lines. Why did authors choose this plant developmental stage for the different evaluations?

Page 13. Even though, the authors did not find differences in the 23S/18S ratio between wild type and hflX mutant lines, still it is possible that the rRNAs were not correctly assembled, and therefore translation was not efficient enough. Chloroplast translation efficiency should be assessed to look for an effect on ribosome reconstitution or functionality.

Reviewer #3:

GTPases assist ribosome assembly, translation, and turnover in prokaryotes and animal cells. Chloroplast have proteins homologous to bacterial HflX proteins have been identified and shown to bind the 50S ribosomal subunit, although their role in ribosome function is unknown. This study focuses on the physiological function of plastid HflX through the generation of T-DNA insertion lines lacking HflX expression and function. HflX was identified as a nuclear encoded gene as well as identifying a second nuclear gene HflX-like. The current study focuses on HflX. The authors demonstrate that interruption of hflx does not seem to be required for normal growth. In addition, through a battery of stresses that are known to affect chloroplast translation (heat shock, cold treatment, high Mn2+, high salt), hflx plants grew comparable to wildtype plants. However, when treated with lincomycin, a drug that interferes with prokaryotic translation by blocking the peptidyl transferase center, the hflx plants were more severely affected than wildtype, suggesting a possible role for HflX in ribosome splitting or recycling. It would have strengthened the manuscript to also include studies on Hflx-like protein as well, but I look forward to seeing characterization of that protein in future work.

Overall, this manuscript is well-written and presents sound experiments in a logical way. I have some

minor issues to be addressed by the authors.

• In the cartoon domain models of cpHflX and E. coli HflX, what does the red region signify? Why is it designated separately from the rest of the NTD?
• Line 227 and Figure S1: please indicate the enlarged N-loop of the NTD of HflX-like protein. It is difficult to distinguish the enlarged N-loop from the N-loops of HflX protein. The images look more like they are at different scales, which would also give the impression that the N-loop of HflX-like is enlarged.
• Figure 2: Can the authors offer an explanation as to why the DNA in hflx1-3 appears to be less than the other lines and wildtype?
• Figure 4C: What about hflx1-3? Did hflx1-3 also show no significant difference between the 23S/18S rRNA ratios?

DECISION LETTER- ROUND 2:

December 7, 2023
Dr. Ben Field
CNRS, CEA, University of Aix-Marseille
BIAM
163 av de Luminy
Marseille, N/A 13009
France

MSID: 2023-01144R1
MS TITLE: Insights into the function of the chloroplastic ribosome-associated GTPase HflX in Arabidopsis thaliana.

Dear Dr. Ben Field:

I am pleased to inform you that your manuscript "Insights into the function of the chloroplastic ribosome-associated GTPase HflX in Arabidopsis thaliana." has been accepted for publication in Plant Direct.

Your article will appear online in the next available issue of Plant Direct. To ensure your article gets published as quickly as possible, please pay attention to the steps detailed below. We have found that most of the delays happen at this stage, especially at the payment stage, so please respond as quickly as possible when prompted.

License Agreement: Once your article has been accepted it will move to Production and undergo admin and file checking - you may receive an email with any queries we have at this stage. When all required items are received by the publisher and queries resolved, the corresponding author will receive an email from Wiley's Author Services system which will ask them to log on at https://authorservices.wiley.com/bauthor and will present them with the appropriate license for completion. Your article cannot be published until both the signed license agreement and payment of the article fee have been received.

Payment of the Open Access Article Publication Fee: All articles published in Plant Direct are fully open access: immediately and freely available to read, download and share. Plant Direct charges a publication fee to cover the publication costs. The corresponding author for this manuscript should have already received a quote from the payments team (cs-openaccess@wiley.com) with the estimated article publication fee; please email cs-openaccess@wiley.com if this has not been received. The corresponding author should log on to the Wiley Author Services site, where the publication fee can be paid by credit card or an invoice. Pro Forma can also be requested. Payment of the publication charge must be received before the article will be published online.

Proofs: You will have the opportunity to look over your paper once more when you receive the author proofs for your article. The proofs will be with you in approximately two weeks. Please note that, in addition to publishing reviewer comments, the author's responses to review comments will also be published alongside the final version of the paper. If you would not like the author's responses to be published, please contact the editorial office at plantdirect@wiley.com .

Promotion of your article: You can help your research get the attention it deserves! Check out Wiley's free Promotion Guide for best-practice recommendations for promoting your work at www.wileyauthors.com/eeo/guide. And learn more about Wiley Editing Services which offers professional video, design, and writing services to create shareable video abstracts, infographics, conference posters, lay summaries, and research news stories for your research at www.wileyauthors.com/eeo/promotion.

Thank you again for your contribution to Plant Direct. If you have any questions, feel free to contact the editorial office at plantdirect@wiley.com .

Sincerely,

David Mendoza-Cozatl

Ana Fortes

Editor, Plant Direct

-- Editor comments

-- Reviewer comments:

AUTHORS' RESPONSE

Responses to reviewers, Mehrez et al. 2023

We would like to thank the reviewers and the editor for their thoughtful consideration of our manuscript. We have now revised the manuscript to take into account the comments raised, and we provide a version of the manuscript with modifications in red.

Comments are numbered, and our replies are marked by a >. We also provide a file containing these responses with the reviewers' comments marked in grey and our response in black.

Editor comments:

One reviewer noted the need for the authors to make sure their claims are supported by the data. This should be thoroughly revised in the next version. Though there is no need to provide additional experiments the statements have to be sound.
> Please see responses 1.3 /1.4 / 1.5

Reviewer comments:
Reviewer #2:

This manuscript reports initial efforts to gather information on the physiological function of the plant HflX protein. With incomplete approaches, the authors address the function of this protein by evaluating the phenotypes of Arabidopsis plants under different growth conditions, including heat, manganese, cold, and salt stress, where they did not detect differences between the null hflX mutants and the wild-type line. However, when they add lincomycin, an antibiotic that affects chloroplast translation, to the plant growth medium, hflX mutants showed resistance to this antibiotic as compared to the wild type. With no further analysis, authors conclude that "HflX does not have a detectable role in plant growth and development, nor does it play a distinct role in acclimation to several different stresses" and "that HflX is required for plant resistance to chloroplast translational stress mediated by the antibiotic lincomycin". Even though the described observations add information to the functional study of this protein in plants, further experimentation is needed to support the concluding statements of this work. Authors should consider the following observations.

Introduction
1.1. How many HflX genes are in Arabidopsis? Does the number of HflX and HflX-like genes vary between different plant species?
> In the introduction we mention the known HflX reported in plants (Suwastika et al., 2014). According to this report there is a single *HFLX* gene in Arabidopsis. HflX-like was identified in the current work, therefore we don't mention it in the introduction.

In our phylogenetic analysis, we selected plants and algae representative of certain groups (monocots, dicots etc) and show all the HflX or HflX-like that are present in the genomes of these plants. All plants analyzed have a single copy of *HFLX* and *HFLX-like*, except for *P. patens* where *HFLX-like* is absent. Although the phylogenetic analysis would suggest that there is usually one of each, we cannot exclude that other plants not tested have different *HFLX* or *HFLX-like* copy numbers. We now mention the observed *HFLX* and *HFLX-like* copy number in the introduction and in the results.

Results
1.2. Page 8. During the analysis of the structural characteristics of the Arabidopsis HflX and HflX-like proteins, did authors look for possible phosphorylation sites given the observation that E. coli HflX apparently possess an autophosphorylation activity (Gosh et al., doi: 10.1002/2211-5463.12065 FEBS 2016)
> Ser211, the site of autophosphorylation of *E. coli* HflX, is conserved in plant HflX and HflX-like. In *C. reinhardtii* HflX-like, the Ser is substituted with a Thr. We now mention this in the results.

1.3. Page 9. After evaluating a rosette growth through the measurement of its size in wild type and mutant lines, authors concluded that HflX does not influence Arabidopsis normal growth and development. However, testing just this phenotype is not enough to reach such wide conclusion. Authors do not mention if they evaluated germination rate, leaf size, flowering time, seed size, yield, root architecture, etc, etc. Authors should assess additional phenotypes during different developmental stages to determine whether there is or not any effect on growth and development in Arabidopsis plants lacking HflX. Maybe checking those traits where an active translation is needed would a good start.

> We agree that our wording is too wide given that we did not quantify a wider range of plant growth parameters. We note that we didn't observe visible differences in germination rate, leaf size (pictured) or flowering time. However, we did not quantify these observations. We also note the rosette growth is usually slower in the presence of chloroplast translation defects (e.g. Meteignier et al. 2021 10.1093/nar/gkaa1244). We have modified the text in the abstract, results and discussion sections to better constrain our claims.

1.4. Page 11. Do authors compare basal heat tolerance of the mutants, or other phenotypes to look at the mutant performance through different heat plant responses? Do authors consider that the evaluation they did is enough to be certain that HflX does not participate in acclimation to HS?

> EcHflX was previously shown to be essential in response to heat-shock in E. coli (Zhang et al. 2015 https://doi.org/10.1038/nsmb.3103). This led us to test a comparable experimental setting in Arabidopsis where we do not observe an essential requirement for HflX. We did try a range of different temperatures and time exposures (not shown) but we always obtained a similar phenotype to the WT.

We are not sure that any scientific result can demonstrate certainty, and this even more challenging in the case of negative results (e.g what about 41oC? Or 40.5? or 40.25? or 12h? etc). Regarding the contribution of HflX we have moved from maybe involved to probably not involved. We have modified the text to make this clearer.

1.5. Page 11. Regarding manganese effects, just one manganese concentration (2 mM) was tested for this evaluation? Why was 2 mM manganese used in these experiments? A manganese concentration sensitivity curve should be done including all lines. Why did authors choose this plant developmental stage for the different evaluations?

> We have found that if we want to find a sensitivity phenotype then it is good to treat plants with a condition that has a clear but not too severe consequence on growth (or other measured parameter). From our own preliminary experiments, and those of other researchers (Zhao et al. 2017 https://doi.org/10.3389/fpls.2017.00272) 2 mM manganese is the lowest concentration that gives a clear phenotype in the wild type. At higher concentrations we might miss potential changes in sensitivity due to the severity of the phenotype.

We used actively growing seedlings for our stress tests because this is when chloroplast biogenesis and translation are particularly active and it is also a convenient growth stage for controlled treatments and phenotype quantification.

1.6. Page 13. Even though, the authors did not find differences in the 23S/18S ratio between wild type and hflX mutant lines, still it is possible that the rRNAs were not correctly assembled, and

therefore translation was not efficient enough. Chloroplast translation efficiency should be assessed to look for an effect on ribosome reconstitution or functionality.

> We agree with the reviewer's comment. We mentioned this point in the discussion section (original line 358-363, revised line 368-373).

Reviewer #3:

GTPases assist ribosome assembly, translation, and turnover in prokaryotes and animal cells. Chloroplast have proteins homologous to bacterial HflX proteins have been identified and shown to bind the 50S ribosomal subunit, although their role in ribosome function is unknown. This study focuses on the physiological function of plastid HflX through the generation of T-DNA insertion lines lacking HflX expression and function. HflX was identified as a nuclear encoded gene as well as identifying a second nuclear gene HflX-like. The current study focuses on HflX. The authors demonstrate that interruption of hflx does not seem to be required for normal growth. In addition, through a battery of stresses that are known to affect chloroplast translation (heat shock, cold treatment, high Mn2+, high salt), hflx plants grew comparable to wildtype plants. However, when treated with lincomycin, a drug that interferes with prokaryotic translation by blocking the peptidyl transferase center, the hflx plants were more severely affected than wildtype, suggesting a possible role for HflX in ribosome splitting or recycling. It would have strengthened the manuscript to also include studies on Hflx-like protein as well, but I look forward to seeing characterization of that protein in future work.

Overall, this manuscript is well-written and presents sound experiments in a logical way. I have some minor issues to be addressed by the authors.

2.1. In the cartoon domain models of cpHflX and E. coli HflX, what does the red region signify? Why is it designated separately from the rest of the NTD?

> We apologize for not explaining the colors in the legend. We used the *E. coli* HflX model presented previously (Zhang et al. 2015 https://doi.org/10.1038/nsmb.3103). The NTD domain contains a nucleotide binding domain marked in blue and a helical domain marked in red. We have modified the legends of Fig. 1 and Fig. S1 accordingly.

2.2. Line 227 and Figure S1: please indicate the enlarged N-loop of the NTD of HflX-like protein. It is difficult to distinguish the enlarged N-loop from the N-loops of HflX protein. The images look more like they are at different scales, which would also give the impression that the N-loop of HflX-like is enlarged.

> We have modified Fig. S1 and Fig. 1 to illustrate the N-loop. We also checked the images and confirm that the scales are identical for the five structure representations in Fig. S1 (the images are produced from a single ChimeraX file containing the aligned 3D structures).

2.3. Figure 2: Can the authors offer an explanation as to why the DNA in hflx1-3 appears to be less than the other lines and wildtype?

> This is a qualitative PCR assay and the difference is likely due to small differences in the starting DNA concentration.

2.4. Figure 4C: What about hflx1-3? Did hflx1-3 also show no significant difference between the 23S/18S rRNA ratios?

> We only tested the mutants *hflx1-1* and *hflx1-2*. As the two mutants showed the same results, we didn't consider it necessary to test *hflx1-3*.
